# Induction of pulmonary antibodies against oxidized lipids in mice exposed to cigarette smoke

**DOI:** 10.1186/s12931-016-0416-6

**Published:** 2016-08-04

**Authors:** Danya Thayaparan, Pamela Shen, Martin R. Stämpfli, Mathieu C. Morissette

**Affiliations:** 1 Medical Sciences Graduate Program, McMaster University, Hamilton, ON Canada; 2 Department of Pathology and Molecular Medicine, McMaster Immunology Research Centre, McMaster University, Hamilton, ON Canada; 3 Department of Medicine, Firestone Institute of Respiratory Health at St. Joseph’s Healthcare, McMaster University, Hamilton, ON Canada; 4 Institut Universitaire de Cardiologie et de Pneumologie de Québec, 2725, Chemin Ste-Foy, G1V 4G5 Quebec City, PQ Canada; 5 Department of Medicine, Faculty of Medicine, Université Laval, Quebec City, PQ Canada

## Abstract

**Background:**

Chronic cigarette smoke exposure is known to activate the adaptive immune system; however, the functional role of these processes is currently unknown. Given the role of oxidized lipids in driving innate inflammatory responses to cigarette smoke, we investigated whether an adaptive immune response against damaged lipids was induced following chronic cigarette smoke exposure.

**Methods and results:**

Using a well-established mouse model, we showed that cigarette smoke exposure led to a progressive increase in pulmonary antibodies against oxidized low-density lipoprotein (OxLDL). Functionally, we found that intranasal delivery of an antibody against oxidized phosphatidylcholine (anti-OxPC; clone E06) increased lipid and particle uptake by pulmonary macrophages without exacerbating cigarette smoke-induced neutrophilia. We also found that anti-OxPC treatment increased particle uptake following smoking cessation. Finally, the frequency of pulmonary macrophages with internalized particles was increased after prolonged smoke exposure, at which time lung anti-OxPC responses were highest.

**Conclusions:**

Altogether, this is the first report to demonstrate a non-pathogenic, and possibly protective, function of a newly identified autoantibody induced by chronic cigarette smoke exposure.

## Background

Cigarette smoking is a complex pulmonary insult causing chronic lung inflammation and lung degenerative diseases such as chronic obstructive pulmonary disease (COPD). A hallmark of advanced COPD, and mice chronically exposed to cigarette smoke, is the formation of tertiary lymphoid tissues (TLTs) in the lungs. These immune structures consist of B and T cells, as well as dendritic cells and macrophages [1–3]. The development of TLTs within the lungs suggests local activation of adaptive immune processes that may generate effector T cells as well as antibody production [4]. In fact, signs of T cell clonal expansion and autoantibody production within the lungs have been identified in humans and animal models [3, 5–9]. However, the role of these adaptive immune processes has yet to be fully understood.

We recently reported that damaged pulmonary lipids play an important role in triggering innate inflammatory responses elicited by cigarette smoke [10]. Characteristic features of cigarette smoke exposure, such as lipid accumulation in macrophages, IL-1α and GM-CSF production, and neutrophil recruitment were recapitulated by delivery of damaged lipids to the lungs [10]. The role of oxidized lipids in inducing inflammatory processes and the accumulation of lipid-laden macrophages are key features shared with atherosclerosis [11–14]. With regard to the adaptive immune system, atherosclerosis is associated with an increase in antibodies against oxidized low-density lipoprotein (OxLDL), largely recognizing the oxidized phospholipid fraction of the macromolecules [15]. Current evidence suggests that these anti-OxLDL antibodies appear to have detrimental as well as beneficial effects on disease pathogenesis [12–15]. As both smoking-induced lung injury and atherosclerosis are driven, in part, by chronic damage to lipids, it is plausible that prolonged cigarette smoke exposure similarly triggers an adaptive response towards oxidized lipids.

We therefore hypothesized that chronic cigarette smoke exposure leads to the production of antibodies against oxidized lipids, and that these antibodies may contribute to limit the magnitude of the response towards damaged pulmonary lipids. To test this hypothesis, we used a well-characterized mouse model of cigarette smoke exposure characterized by rapid and persistent activation of innate immune processes, followed by induction of adaptive immune responses [10, 16, 17]. We found that chronic exposure to cigarette smoke led to the induction of antibodies against oxidized LDL in the lungs. Moreover, delivery of a mouse monoclonal antibody against oxidized phosphatidylcholine (OxPC) during acute smoke exposure reduced some inflammatory markers, and increased lipid and particle uptake by pulmonary macrophages. Altogether, this is the first report to document the presence of antibodies against oxidized LDL following chronic smoke exposure and its link to a non-pathogenic, and possibly protective, function in the lungs.

## Methods

### Cigarette smoke exposure and interventions

Six to 8 week old female C57BL/6, BALB/c, and A/J mice were exposed to cigarette smoke using a well-characterized whole-body exposure system for 1 h, twice a day, 5 days per week for up to 24 weeks [10, 16, 17]. Control groups were exposed to room air. The Animal Research Ethics Board of McMaster University approved all experimental procedures (Animal Utilization Protocol 07-09-57).

A monoclonal IgM antibody against oxidized phosphatidylcholine (clone E06, Avanti Polar Lipids, Alabaster, AL, USA) or a mouse IgM isotype control (clone MM-30, Biolegend, San Diego, CA, USA), was delivered intranasally (20 μg in 35 μl of sterile PBS) every day 1 h prior to the first cigarette smoke exposure, or at a similar time for cessation experiments.

### Assessment of bronchoalveolar lavage cells and mediators

Mice were anesthetized with isoflurane and euthanized by exsanguination. Lungs were removed from the chest cavity and the trachea canulated. Bronchoalveolar lavage (BAL) was performed by lavaging the lungs twice with 500 μl of sterile cold PBS. Total cell concentration was determined using a hemacytometer. The BAL was then centrifuged at 800 g for 8 min. Cytospins were prepared from the resuspended cell pellet and the differential counts performed by counting at least 300 cells per cytospin. Levels of monocyte chemoattractant protein-1 (MCP-1), granulocyte-macrophage colony-stimulating factor (GM-CSF), and interleukin 1 alpha (IL-1α) were measured in the BAL fluid by ELISA according to the manufacturers’ instructions (MCP-1 and IL-1 α: R&D Biosystems, Minneapolis, MN, USA; GM-CSF: eBioscience Inc., San Diego, CA, USA).

### Measurement of antibodies with affinity for oxidized LDL

Antibodies against oxidized LDL (OxLDL) were measured in the mouse BAL fluid. Nunc-immuno Multisorp plates were coated overnight at 4 °C with 2 μg of human OxLDL (AlfaAesar, Heysham, UK) per well in 100 μl of PBS. Wells were washed 3 times with PBS-T and then blocked with PBS-T-BSA (PBS; 0.05 % Tween 20; 5 % bovine serum albumin) for 1 h at room temperature. Samples diluted in PBS-T-1 % BSA were incubated for 2 h at room temperature on an orbital plate shaker at 550 rpm. Wells were washed four times with PBS-T. For total anti-OxLDL assessment, wells were incubated with a rabbit anti-mouse Ig (H + L chains) coupled to HRP (Sigma-Aldrich Canada, Oakville, ON, Canada) for 1 h. For isotype-specific assessment, wells were incubated with a biotinylated rabbit anti-mouse IgM, a biotinylated rabbit anti-mouse IgA, or a rabbit anti-mouse IgG for 1 h, followed by a 30-minute incubation with streptavidin-HRP (Sigma-Aldrich Canada, Oakville, ON, Canada). HRP substrate was used and the absorbance at 550 nm measured (Bio-Rad, Hercules, CA, USA).

### Assessment of pulmonary macrophage size, intracellular lipids, and particle content

Pulmonary macrophage size and intracellular lipids were assessed as previously described using BAL cytospins [10]. Particle inclusions in macrophages were also assessed using BAL cytospins. Black carbon-like inclusions were counted in 300 to 400 macrophages/mouse. Data are expressed as percentage of macrophages with 0, 1–4, and ≥5 particle inclusions, or as the absolute number of particle-containing macrophages (see figure legends for details).

### Statistical analysis

Statistical differences were assessed using unpaired Student’s *t*-test (2 groups) or one-way ANOVA (>2 groups) followed by a post-hoc comparison of every group using a Bonferroni correction for multiple comparisons. Tests were performed using GraphPad Prism.

## Results

### Chronic cigarette smoke exposure induces pulmonary antibodies against oxidized LDL

**Fig. 1:**
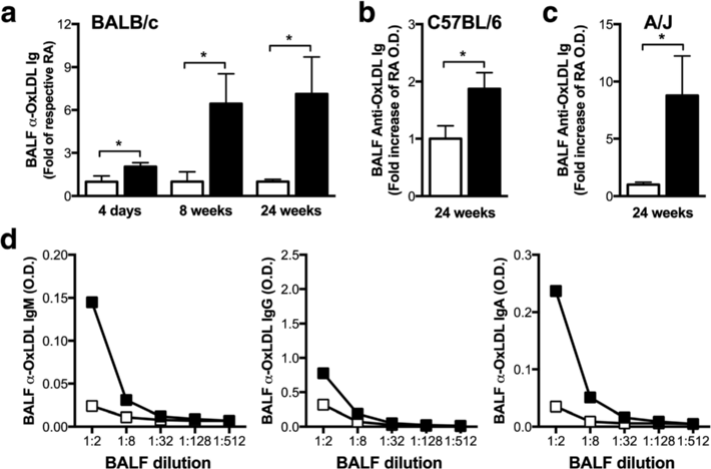
Cigarette smoke exposure leads to an increase in pulmonary antibodies with affinity for oxidized LDL. Female 6–8 week old mice were exposed to room air (RA) or cigarette smoke (CS) for 1 h, twice a day, 5 days a week for 4 days, 8 weeks, or 24 weeks. Levels of total immunoglobulins with affinity for oxidized low-density lipoprotein (OxLDL) were measured by direct ELISA in the bronchoalveolar lavage fluid (BALF) of **a** BALB/c, **b** C57BL/6, and **c** A/J mice. **d** Isotypes were measured by ELISA in the BALF of BALB/c mice exposed for 24 weeks to RA or CS using isotype specific secondary antibodies. Data represent mean ± SEM; *n* = 4–5/group; *p < 0.05; ***p < 0.001

To investigate whether persistent cigarette smoke exposure elicits antibody responses against oxidized lipids, we exposed BALB/c mice to cigarette smoke for 4 days, 8 weeks, and 24 weeks. Anti-OxLDL antibodies were assessed by ELISA in the bronchoalveolar lavage fluid (BALF) and the serum. As controls, we included age-matched, room air-exposed mice in the analysis. We found that BALF levels of anti-OxLDL antibodies increased over time to reach levels 6-fold higher than room air controls after 8 and 24 weeks of cigarette smoke exposure (\). Elevated levels of anti-OxLDL antibodies were also observed in C57BL/6 and A/J mice exposed for 24 weeks to cigarette smoke (Fig. 1b-c). Similar to our observations with anti-nuclear autoantibodies, we observed a more robust increase in BALB/c than in C57BL/6 mice [3]. Of note, we did not observe increased levels of anti-OxLDL antibodies in the serum of cigarette smoke-exposed mice at any of the time points included in the analysis (data not shown). Isotype analyses in BALB/c mice revealed marked increases in IgM and IgA anti-OxLDL antibodies following exposure to cigarette smoke for 24 weeks, with only a small increase in IgG (Fig. 1d). These data show for the first time that cigarette smoke exposure elicits antibodies against oxidized lipids.

### Antibodies against oxidized phosphatidylcholine do not exacerbate cigarette smoke-induced lung inflammation

**Fig. 2:**
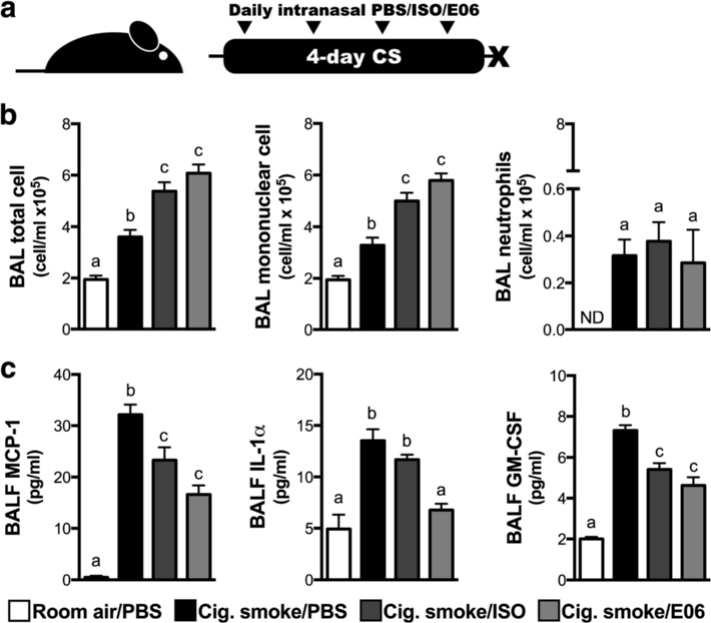
Impact of anti-OxPL antibody delivery on CS-induced pulmonary inflammation. **a** Female 6–8 week old C57BL/6 mice were exposed to room air (RA) or cigarette smoke (CS) for 1 h, twice a day for 4 days. They received phosphate-buffered solution (35 μl; PBS), a mouse IgM isotype control (20 μg in 35 μl; ISO), or a mouse monoclonal antibody against oxidized phosphadytilcholine (20 μg in 35 μl; clone E06) intranasally every day before the first cigarette smoke exposure of the day. **b** Total cells, mononuclear cells and neutrophils were counted in the bronchoalveolar lavage (BAL). **c** Monocyte chemotactic protein 1 (MCP-1), interleukin-1 alpha (IL-1α), and granulocyte macrophage colony-stimulating factor (GM-CSF) levels were measure in the BAL fluid (BALF) by ELISA. Data represent mean ± SEM; *n* = 4–5/group; representative of two independent experiments; bars with different superscripts are significantly different from each other

Since cigarette smoke exposure elicited the production of antibodies against oxidized phospholipids, we next investigated whether these antibodies impact smoke-induced inflammatory processes, favoring a short-term exposure protocol to minimize the amount of endogenously produced anti-OxLDL antibodies in cigarette smoke-exposed mice. To this end, we exposed mice to cigarette smoke for 4 days and administered daily a monoclonal antibody against oxidized phosphatidylcholine (OxPC), the most prevalent phospholipid in lung surfactant. We found that the anti-OxPC had no effect on the cellular response to smoke exposure when compared to isotype control (Fig. 2b). Both isotype and anti-OxPC antibodies lead to an increase in mononuclear cells (Fig. 2b). Interestingly, IL-1α, a key mediator in the pulmonary response to cigarette smoke exposure [10, 18], was significantly reduced in the anti-OxPC-treated compared to the isotype control group. In contrast, MCP-1 and GM-CSF levels were similar between the two experimental groups (Fig. 2c). Altogether, these data suggest that antibodies against OxPC do not exacerbate cigarette smoke-induced inflammatory processes.

### Antibodies against oxidized phosphatidylcholine promote lipid and particle uptake by pulmonary macrophages

**Fig. 3:**
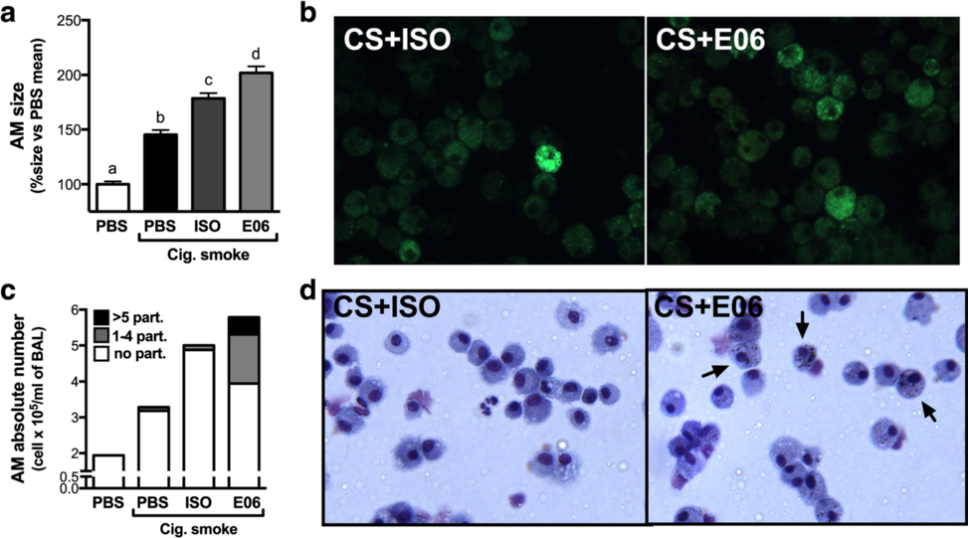
Anti-OxPL antibodies increase lipid and smoke particle uptake by pulmonary macrophages. Female 6–8 week old C57BL/6 mice were exposed to room air (RA) or cigarette smoke (CS) for 1 h, twice a day for 4 days. They received phosphate-buffered solution (35 μl; PBS), a mouse IgM isotype control (20 μg in 35 μl; ISO), or a mouse monoclonal antibody against oxidized phosphatidylcholine (20 μg in 35 μl; clone E06) intranasally each day before the first cigarette smoke exposure of the day. Bronchoalveolar lavage (BAL) cytospins were used to assess **a** the size of pulmonary macrophages and **b** were also stained with the lipophilic dye BODIPY to detect intracellular lipid accumulation in macrophages. **c** Black particles were counted in pulmonary macrophages and expressed as the absolute number of macrophages with no (*white*), 1–4 (*grey*) or >5 (*black*) intracellular particles. **d** Representative BAL cells cytospins of cigarette smoke-exposed and PBS or anti-OxPC (E06)-treated animals with arrows showing macrophages with internalized smoke particles. Data represent mean ± SEM; *n* = 4/group; representative of two independent experiments. Bars with different superscripts are significantly different from each other

Pulmonary macrophages are key orchestrators of the pulmonary response to cigarette smoke exposure [10, 19]. We therefore investigated the effect of anti-OxPC antibody delivery during smoke exposure on pulmonary macrophages. We observed that anti-OxPC delivery led to an increase in the size of pulmonary macrophages, which appeared to be largely driven by an increased accumulation of intracellular lipid droplets (Fig. 3a-b). We also noticed that anti-OxPC treatment markedly increased the uptake of black carbon-like particles, likely particles contained within cigarette smoke (Fig. 3c-d). These data suggest that antibodies against OxPC have the ability to increase lipid uptake by macrophages and facilitate the internalization of cigarette smoke particles.

### Antibodies against oxidized phosphatidylcholine promote particle uptake following cessation of cigarette smoke exposure

**Fig. 4:**
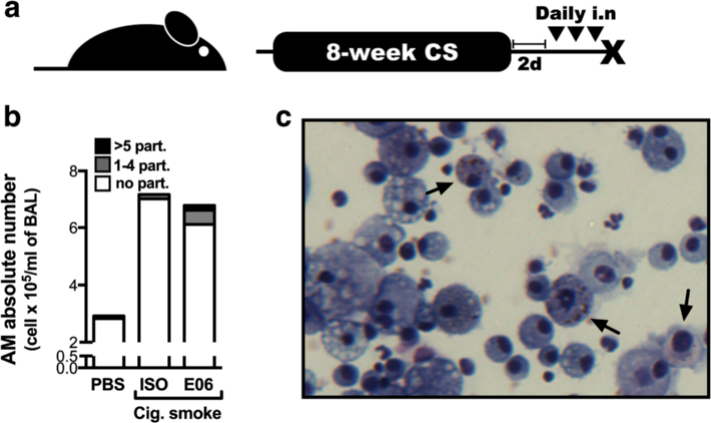
Anti-OxPL antibodies increase smoke particle uptake by pulmonary macrophages when delivered during smoking cessation. **a** 6–8 week old female C57BL/6 mice were exposed to room air (RA) or cigarette smoke (CS) for 1 h, twice a day, 5 days a week for 8 weeks. They were then subjected to 2 days of complete smoking cessation and, on the morning of the following 3 consecutive days, received phosphate-buffered solution (35 μl; PBS), a mouse IgM isotype control (20 μg in 35 μl; ISO), or a mouse monoclonal antibody against oxidized phosphadytilcholine (20 μg in 35 μl; clone E06) intranasally. **b** Black smoke particles were counted in pulmonary macrophages and expressed as the absolute number of macrophages with no (*white*), 1–4 (*grey*) or >5 (*black*) intracellular particles. **c** A representative cytospin from cigarette smoke-exposed mice that received the E06 antibody during cessation

When delivered daily during a short-term cigarette smoke exposure protocol, the anti-OxPC antibody caused smoke particles to be internalized by pulmonary macrophages. As particles can remain trapped in the lungs for years after smoking cessation, we sought to investigate if administration of the anti-OxPC antibody after smoking cessation could facilitate the internalization of particles trapped in the lungs. Mice were exposed to cigarette smoke for 8 weeks and, following two days of smoking cessation, received anti-OxPC or isotype control antibodies intranasally for 3 consecutive days (Fig. 4a). Anti-OxPC antibody delivery markedly increased the number of internalized smoke particles in alveolar macrophages (Fig. 4b-c). This suggests that the anti-OxPC antibodies have the ability to promote the uptake of particles within the lung by pulmonary macrophages following smoking cessation.

### Particle uptake by pulmonary macrophages increases over the course of exposure to cigarette smoke

**Fig. 5:**
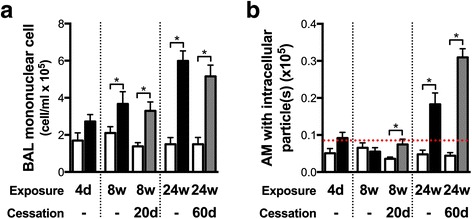
The proportion of pulmonary macrophages with intracellular particles increases over the course of cigarette smoke exposure. Female 6–8 week old BALB/c mice were exposed to room air (RA) or cigarette smoke (CS) for 1 h, twice a day, 5 days a week for 4 days, 8 weeks, or 24 weeks, and followed by cessation periods. **a** Lungs were subjected to bronchoalveolar lavage (BAL), total cells counted, and cell differentials performed to determined the absolute number of mononuclear cells in the BAL. **b** Black particles were counted in pulmonary macrophages and expressed as the absolute number of macrophages with intracellular particles. The dashed red line represents the highest number of pulmonary macrophage with internalized particles observed in a room air exposed mouse (particle background). Data represent mean ± SEM; *n* = 4–5/group; *p < 0.05

As anti-OxPC antibodies promote particle uptake, we sought to investigate if the frequency of macrophages with internalized particles increased over time, mirroring the humoral response to oxidized lipids in cigarette smoke-exposed mice. We found that the number of mononuclear cells in the BAL increased with the duration of cigarette smoke exposure (Fig. 5a). The number of pulmonary macrophages with internalized particles only increased above baseline after 24 weeks of cigarette smoke exposure and remained elevated following smoking cessation (Fig. 5b). These observations suggest that particles accumulate in macrophages over a prolonged period of smoke exposure or, alternatively, that antibodies against oxidized lipids contribute to increase particle uptake at these time points.

## Discussion

We previously showed that cigarette smoke exposure disrupts pulmonary lipid homeostasis [10], leading to innate immune activation. In the present study, we investigated whether persistent innate immune activation induces humoral immune responses against oxidized lipids, and the consequences of these antibody responses to cigarette smoke-induced inflammatory processes.

Using a well-characterized pre-clinical model of cigarette smoke exposure [3, 10], we investigated the levels of antibodies against oxidized lipids in the bronchoalveolar lavage and serum of mice exposed to cigarette smoke. Antibodies were measured by ELISA, using plates coated with OxLDL, a rich source of oxidized lipids. We observed a time-dependent increase in anti-OxLDL antibody levels in the BAL of BALB/c mice with a small, albeit, statistically significant increase after 4 days of smoke exposure. The increase was most pronounced after 8 and 24 weeks. Of note, anti-OxLDL antibodies were increased in the lungs but not the serum. This observation is in agreement with previous reports from our laboratory, documenting local production of anti-nuclear antibodies following cigarette smoke exposure for 8 and 24 weeks [3] and suggests that antibodies were likely produced locally. It is possible that the induction of autoantibodies in response to cigarette smoke exposure, including anti-OxLDL and anti-nuclear antigen antibodies [3], are part of a response aimed at clearing pro-inflammatory damage-associated molecular patterns in the lungs.

The origin of anti-OxLDL antibodies after 4 days of smoke exposure is currently not understood, as *de novo* induction of B cell responses takes several days. Anti-OxLDL antibodies are naturally found in the circulation, even under homeostatic conditions, and have been shown to aid in the clearance of apoptotic cells and bacterial agents [20, 21]. Hence, it is possible that the early increase in anti-OxLDL antibodies after 4 days of smoke exposure is secondary to smoke-induced vascular leakage and reflects a non-specific increase in serum antibodies in the lungs. Alternatively, germline-encoded natural antibodies produced by B1 cells target a diverse range of antigens and, although the spontaneous production of natural antibodies remains poorly understood, specific idiotypes have been shown to be increased in the context of infection or chronic disease [20, 22, 23]. Thus, it is possible that acute exposure to cigarette smoke induces B cells within the lung to release antibodies with specificity for OxLDL as part of an innate inflammatory response independent of TLT formation. Further research is required to determine the precise origin of the anti-OxLDL antibodies observed in our model.

The predominant isotypes of anti-OxLDL measured in the BAL were IgM and IgA. IgA is a defining feature of mucosal B cell responses, suggesting that chronic exposure to cigarette smoke induced a lung-specific humoral response. Moreover, we reported a similar pattern with anti-nuclear antibodies (ANA) in cigarette smoke-exposed mice, which also showed a time dependent induction with a preferential increase in IgM and IgA isotypes [3].

Functionally, anti-OxPC antibodies significantly increased lipid and particle uptake by pulmonary macrophages. Phospholipids are abundant in the pulmonary surfactant, and in the context of cigarette smoke exposure these lipids are prone to be oxidized. The observed increased lipid uptake by pulmonary macrophages may be the result of antibody-mediated aggregation of damaged lipids. Similarly, smoke particles can interact with and get trapped in the pulmonary surfactant [24, 25]. Anti-OxPC antibodies may aggregate smoke particles coated with oxidized surfactant, thus facilitating their uptake by macrophages. Interestingly, the particle uptake was observed following smoking cessation suggesting that anti-OxPC antibody-mediated uptake does not require active smoking, and can likely facilitate the elimination of previously trapped smoke particles. These findings are similar to what we observed following prolonged smoke exposure where increased levels of anti-OxLDL antibodies are associated with increased numbers of particle-containing macrophages. These antibodies could therefore help in the clearance of smoke particles by pulmonary macrophages, and appear to play a protective role. This may provide an opportunity to develop therapeutic strategies to clear particles trapped in the lung; however, further research is required to show this conclusively.

Increased lipid and particle uptake by anti-OxPC antibodies did not exacerbate the inflammatory processes in cigarette smoke-exposed mice. Despite an increase in mononuclear cells, which was also observed in the isotype-treated group, no further increase in neutrophils was observed. Moreover, we observed decreased levels of IL-1α, a critical mediator of cigarette smoke-induced inflammation, in the anti-OxPC-treated group. Therefore, these antibodies could help clear damaged lipids and smoke particles from the lungs without aggravating the inflammatory response. Of note, damaged or oxidized lipids, and smoke particles are potentially more damaging or pro-inflammatory when left in the extracellular milieu than when internalized. Oxidation of phospholipids can lead to the enzymatic release of lipid species with ‘platelet-activating factor’ (PAF)-like activity and lysophospholipids that have well-documented pro-inflammatory effects [26–28]. Smoke particles can also contain heavy metals such as iron, which catalyze the generation of ‘reactive oxygen species’ (ROS) through the Fenton reaction [29]. Further investigation is required to fully understand the mechanisms by which anti-OxPC antibodies alter the pulmonary inflammatory response to cigarette smoke.

## Conclusions

While markers of B cell activation are increased in COPD/emphysema, as well as in mice chronically exposed to cigarette smoke [1–3, 30, 31], the role of B cells in the pathogenesis of COPD remains poorly understood. Whether these B cell-mediated responses are beneficial or detrimental will likely depend on antigen specificity as well as isotype. This study describes for the first time the pulmonary humoral response to oxidized lipids induced by cigarette smoke exposure. It also provides insight into the potential role of these antibodies in the general pulmonary response to cigarette smoke. Additional investigations will be necessary to fully appreciate the role of the humoral response to damaged lipids in the context of cigarette smoke exposure, and its potential as a therapeutic tool.
